# Prevalence and Risk Indicators of Peri‐Implant Diseases and Buccal Soft‐Tissue Dehiscence: A Cross‐Sectional Study From a University‐Based Cohort

**DOI:** 10.1111/jre.70025

**Published:** 2025-08-07

**Authors:** Giacomo Baima, Federica Romano, Sompol Chuachamsai, Marta Ciccarelli, Andrea Lo Giudice, Marco Ventricelli, Giulia Maria Mariani, Mario Romandini, Gianmario Schierano, Mario Aimetti

**Affiliations:** ^1^ Department of Surgical Sciences, C.I.R. Dental School University of Turin Turin Italy; ^2^ Perio‐Implant Innovation Center, Institute for Integrated Oral, Craniofacial and Sensory Research–National Clinical Research Center of Stomatology, Ninth People's Hospital Shanghai Jiao Tong University School of Medicine Shanghai China

**Keywords:** dental implants, epidemiologic factors, implant loss, keratinized tissue, malposition, peri‐implant recessions, peri‐implantitis, periodontal diseases, prevalence, risk factors

## Abstract

**Aim:**

To assess the prevalence of peri‐implant diseases and buccal peri‐implant soft‐tissue dehiscence (PISTD) and to identify the associated risk indicators.

**Methods:**

Patients previously rehabilitated with implant‐supported rehabilitations at the University of Turin Dental School were specifically recalled with a registry‐based approach for this cross‐sectional study. Data collection included medical and dental history, full‐mouth clinical examination, and periapical radiographs. Moderate/severe peri‐implantitis was diagnosed based on bone loss (direct criterion, when available) or bone level (indirect criterion) ≥ 2 mm and the presence of bleeding/suppuration. PISTD was defined as mucosal dehiscence on the buccal aspect of at least one implant site. Multilevel, multivariable logistic regression models were applied to identify factors associated with moderate/severe peri‐implantitis and buccal PISTD.

**Results:**

Of the 397 patients contacted, 146 were included (mean age 61.1 ± 14.5 years; current smokers 34.3%; stage III‐IV periodontitis 65.1%) with a total of 511 dental implants (mean function time: 13.3 years [2–31]). Implant survival rate was 96.5%. Moderate/severe peri‐implantitis was detected in 56.8% of patients and 34.7% of implants. Prevalence of buccal PISTD was 54.1% and 40.5%, respectively. Protective indicators for moderate/severe peri‐implantitis included supportive peri‐implant care > twice a year (OR = 0.16; 95% CI: 0.03–0.95), > 2 mm of keratinized tissue height (OR = 0.44; 95% CI: 0.21–0.95), and correct mesio‐distal implant positioning (OR = 0.54; 95% CI: 0.32–0.94). Risk indicators included stage III–IV periodontitis (OR = 2.82; 95% CI: 1.30–6.15), function time ≥ 10 years (OR = 3.02; 95% CI: 1.55–5.89), bisphosphonate use during follow‐up (OR = 5.96; 95% CI: 1.33–26.66), and presence of a cantilever (OR = 5.51; 95% CI: 1.56–19.38). For PISTD, protective indicators were mandibular location (OR = 0.45; 95% CI: 0.25–0.81), thick buccal soft‐tissue phenotype (OR = 0.18; 95% CI: 0.08–0.42), and > 2 mm of keratinized tissue height (OR = 0.05; 95% CI: 0.02–0.15). Risk indicators included peri‐implantitis (OR = 2.21; 95% CI: 1.25–3.91), use of intermediate abutments (OR = 4.92; 95% CI: 1.92–12.58), and proximity to adjacent implants (OR = 3.35; 95% CI: 1.50–7.48) or edentulous spaces (OR = 3.38; 95% CI: 1.51–7.54).

**Conclusion:**

In this long‐term, university‐based cohort, peri‐implant diseases and PISTD were highly prevalent. Multiple patient‐ and implant‐level factors emerged as significant risk or protective indicators. Despite the widespread occurrence of peri‐implant diseases, long‐term implant survival remained high, challenging current diagnostic thresholds and underscoring the need for refined, progression‐based definitions.

## Graphical abstract

This cross‐sectional study of 146 patients and 511 implants assessed the prevalence and risk indicators of peri‐implant diseases and buccal soft‐tissue dehiscence (PISTD) in a university‐based cohort. Moderate/severe peri‐implantitis affected 34.7% of implants, and PISTD was observed in 54.1% of patients. Risk indicators for peri‐implantitis included advanced periodontitis, longer implant function time, bisphosphonate use, and cantilevers. PISTD was associated with peri‐implantitis, intermediate abutments, and proximity to adjacent implants or edentulous spaces. Despite high disease prevalence, implant survival remained high, emphasizing the need for refined, progression‐based diagnostic thresholds.

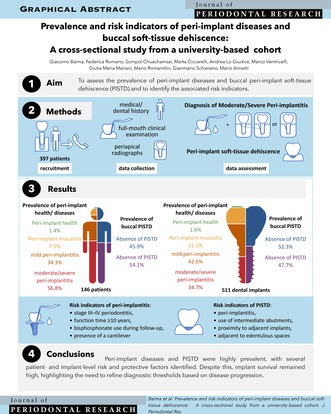

**Summary**

- Background
  ○ Peri‐implant diseases and buccal soft‐tissue dehiscence (PISTD) are frequent biological complications in implant dentistry; yet their long‐term prevalence and associated risk indicators remain incompletely defined, particularly in university‐based populations.
- Added value of this study
  ○ This large cross‐sectional study employed both direct and indirect diagnostic criteria to evaluate the prevalence of peri‐implantitis and PISTD in a university‐based cohort. Using multilevel multivariate models, it corroborates and refines findings from previous studies by identifying key patient‐ and implant‐level risk and protective indicators, including periodontitis diagnosis, implant function time, prosthetic design, soft‐tissue characteristics, and supportive peri‐implant care.
- Clinical implications
  ○ The results emphasize the importance of individualized risk assessment and supportive care in preventing peri‐implant complications. They also underline the need to refine diagnostic thresholds and adopt longitudinal monitoring strategies to better identify and manage progressive disease forms.

## 1 Introduction

Peri‐implantitis is an inflammatory disease affecting the tissues surrounding dental implants, characterized by mucosal inflammation and progressive bone loss, ultimately threatening implant survival [1]. Reported prevalence rates are high but vary widely due to heterogeneous populations and differing disease definitions, particularly regarding bone loss thresholds [2, 3, 4, 5].

Despite the availability of several nonsurgical and surgical treatment modalities, achieving long‐term resolution of peri‐implantitis remains challenging, with frequent disease recurrence [6, 7, 8, 9, 10, 11, 12, 13, 14]. This highlights the need for effective primary prevention strategies targeting modifiable risk factors. However, despite increasing research efforts, only a limited number of risk and protective indicators have been conclusively established [15, 16, 17].

Many previous studies have indeed relied on convenience samples (e.g., patients in supportive peri‐implant care programs) or employed indirect diagnostic criteria (e.g., bone levels instead of direct bone loss assessments) [1], potentially limiting the accuracy of prevalence estimates and risk factor identification [18, 19, 20]. The use of baseline radiographs is essential to ensure diagnostic accuracy, as heterogeneous amounts of early bone remodeling may otherwise be misclassified as disease and vice versa [17, 21, 22]. Furthermore, most epidemiological investigations have focused on implants with ≤ 10 years of function [19, 20], potentially underestimating the true burden of disease [3].

In addition to peri‐implantitis, peri‐implant soft‐tissue dehiscence (PISTD) represents a clinically relevant complication, being frequently associated with esthetic concerns [23]. However, the prevalence, etiology, and potential association of PISTD with peri‐implantitis remain insufficiently characterized [24, 25]. The present university‐based cross‐sectional study aims to address these gaps by assessing the prevalence of peri‐implantitis and PISTD using both direct and indirect diagnostic criteria. This investigation additionally provides an analysis of associated risk and protective indicators at both patient‐ and implant‐level.

## 2 Methods

This manuscript adheres to the STrengthening the Reporting of OBservational studies in Epidemiology (STROBE) guidelines [26]. The study was conducted in accordance with the Declaration of Helsinki for human studies, and its research protocol was ethically approved (protocol no. 17704, dated 15/02/2022) by the Ethical Committee of A.O.U. Città della Salute e della Scienza, Turin, Italy.

### 2.1 Population

This observational study was conducted at the C.I.R. Dental School, University of Turin, Italy, between February 2022 and July 2024. Eligible participants comprised all patients who had received implant treatment at the Sections of Prosthodontics and Periodontics that were rehabilitated by the same experienced prosthodontist (G.S.) between 1993 and 2022, as documented in a continuously maintained clinical track list. To minimize selection bias, patients were specifically recalled with a registry‐based approach for this cross‐sectional study and no exclusion criteria were applied other than the presence of at least one implant in function for a minimum of 2 years at the time of examination to exclude early failures.

Following prosthetic rehabilitation, all patients had received personalized instructions regarding the need for supportive peri‐implant care. However, due to logistic and administrative constraints, not all individuals were enrolled in a structured university‐based SPIC program. Some patients continued to receive follow‐up care at the hospital, while others sought SPIC elsewhere or did not attend regular supportive therapy. For the purpose of this study, patients were contacted using a standardized telephone protocol. In the case of initial nonresponse, at least five additional contact attempts were made on different days and times before classifying a subject as unreachable.

### 2.2 Exposures and Comparisons

#### 2.2.1 History and Dental Record Collection

A trained examiner (S.C.) conducted structured interviews to obtain data on demographics, smoking and alcohol consumption, systemic conditions (e.g., diabetes, osteoporosis, and cardiovascular diseases), and current medications/supplements (e.g., bisphosphonates [either oral or intravenous], proton pump inhibitors, antidepressant, and vitamin D). Additional information included prior periodontal treatments, reasons for tooth loss at implant sites, and frequency of supportive peri‐implant care (SPIC; no/sporadic, once/year, twice/year, and >twice/year). All information was cross‐verified with available track records.

Patient records were further reviewed for detailed implant and prosthetic data, including implant brand, connection type, and platform configuration. Prosthetic details encompassed retention mode, unit, and type. The timing of implant placement, loading, any soft/hard tissue augmentation, years in function, and complications (type and timing) were also recorded if available.

#### 2.2.2 Clinical Examination

Collected data included the number of teeth and implants, Full Mouth Bleeding Score (FMBS), Full Mouth Plaque Score (FMPS), and the number of periodontal sites with PPD ≥ 4 mm with bleeding on probing (BoP+) and ≥ 6 mm. Periodontal status was classified at the examination time according to the 2017 World Workshop staging (I–IV), grading (A–C), and extent (localized/generalized) [27].

All study implants were assessed at six sites per implant for PPD, PISTD, plaque, BoP, suppuration (SoP), and keratinized tissue height using a PCP‐UNC15 periodontal probe, with readings rounded to the nearest millimeter. PISTD was measured in millimeters at the mid‐buccal aspect, as the vertical distance from the mucosal margin to the crown–abutment junction. Calibration was conducted using repeated measurements in 20 patients with 45 implants not included in the study. Measurements were performed independently by two trained examiners (S.C. and M.C.), blinded to each other's results, and repeated by each examiner after a 7‐day interval under identical conditions. All readings were rounded to the nearest millimeter. The intra‐class correlation coefficients (ICCs) for inter‐examiner reliability were 0.82 for PPD and 0.91 for PISTD. The intra‐examiner ICCs were 0.87 and 0.93 for PPD, while 0.94 and 0.91 for PISTD, indicating excellent reproducibility for both parameters.

Buccal soft‐tissue phenotype was categorized as thin (< 1 mm) or thick (> 1 mm) based on probe visibility [28]. Implant positioning (vestibular‐oral) was evaluated clinically based on prosthetic contour and screw access. Interdental hygiene access was assessed using a probe to verify clearance for oral hygiene devices [5].

#### 2.2.3 Radiographic Examination

Periapical radiographs were obtained during the follow‐up visit, with radiographic angulation standardized using the long‐cone paralleling technique. Historical radiographs were additionally retrieved from key timepoints (implant placement, loading, 1 year after loading, and previous follow‐ups). Analog films were digitized using a DSLR camera (Canon EOS 77D, 100 mm macro lens) and light board to preserve image clarity. All collected radiographs were assessed by a single calibrated investigator (S.C.) using the software ImageJ (Wayne Rasband, National Institutes of Health, Bethesda, MD, USA). Calibration used known implant length or inter‐thread pitch. Mesial and distal peri‐implant bone levels were measured from the intra‐osseous implant portion to the first bone contact; the highest value was used for analysis. Angles at the abutment–prosthesis and implant–abutment junctions were measured mesially and distally; the largest values were recorded. Additional assessments included abutment type (straight/angled), implant–abutment diameter match, presence of misfit (implant–abutment and abutment–prosthesis), and mesio‐distal distances to adjacent teeth or implants. Reference points were the implant shoulders and the closest point of the adjacent root or implant. Distances < 1.5 mm to teeth or < 3 mm to implants were considered suboptimal. One hundred radiographs were remeasured after 1 month to ensure consistency; ICC for marginal bone level was 0.84 (95% CI: 0.81–0.88).

### 2.3 Outcomes

#### 2.3.1 Peri‐Implant Health and Diseases

Case definitions for peri‐implant health and diseases were as follows:

- Peri‐implant health: absence of BoP/SoP, without bone loss (< 0.5 mm) (direct evidence) or bone level < 1 mm (indirect evidence).
- Peri‐implant mucositis: presence of BoP/SoP at ≥ 1 site, without bone loss (< 0.5 mm) (direct evidence) or bone level < 1 mm (indirect evidence).
- Peri‐implantitis: presence of BoP/SoP in ≥ 1 site, with bone loss (≥ 0.5 mm) (direct evidence) or bone level ≥ 1 mm (indirect evidence).

These were considered “mixed criteria,” as direct evidence was prioritized whenever baseline radiographs were available; otherwise, indirect evidence was applied. Direct evidence of bone loss was determined by subtracting bone levels at baseline from those measured in follow‐up radiographs. Mesial and distal levels were determined separately, and the worst value was taken for analyses. Baseline radiographs were ideally the ones collected 1 year after loading; in their absence, those obtained at prosthetic loading (second preference) were used.

The severity of peri‐implantitis was further stratified using the same diagnostic hierarchy:

- Mild: bone loss ≥ 0.5 and < 2 mm, or bone level ≥ 1 and < 2 mm.
- Moderate: bone loss ≥ 2 and < 3 mm, or bone level ≥ 2 and < 3 mm.
- Severe: bone loss ≥ 3 mm, or bone level ≥ 3 mm.

The correspondence between direct and indirect thresholds was based on a previous diagnostic accuracy study [21].

As sensitivity case definitions, inflammation was considered using an alternative BoP/SoP threshold (≥ 4 sites instead of ≥ 1), and peri‐implantitis was additionally defined using indirect evidence criteria from the VIII European Workshop on Periodontology [18] and 2017 World Workshop (presence of BoP/SoP in ≥ 1 site, bone level ≥ 3 mm, and PPD ≥ 6 mm) [1].

#### 2.3.2 Peri‐Implant Buccal Soft‐Tissue Dehiscence

**FIGURE 1:**
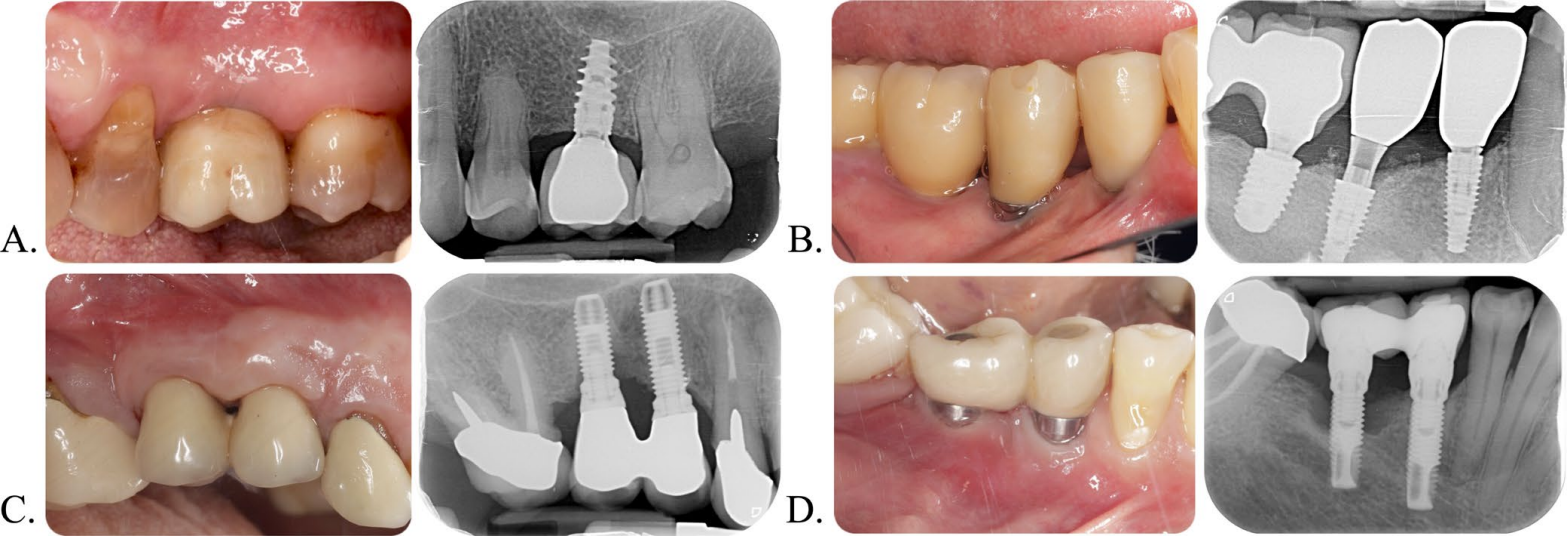
Clinical and radiographic examples of peri‐implant tissue conditions. (A) Healthy implant site without buccal soft‐tissue dehiscence. (B) Healthy implant site with buccal soft‐tissue dehiscence. (C) Implant affected by peri‐implantitis without buccal soft‐tissue dehiscence. (D) Implant affected by peri‐implantitis with buccal soft‐tissue dehiscence.

PISTD was measured in millimeters when there was exposure of the prosthetic abutment, implant neck, or implant surface [24, 29]. For the present study, only buccal PISTD was considered, defined as the presence of a dehiscence in at least one buccal site. PISTD was reported according to different cutoffs (> 0 mm, > 1 mm, > 2 mm). A sensitivity analysis was also conducted only for implants not affected by peri‐implantitis. Figure 1 shows representative cases of both peri‐implant health and peri‐implantitis, with and without the presence of PISTD.

### 2.4 Sample Size

Although the study population was predetermined based on the available clinical registry, a post hoc sample size estimation was conducted to assess the adequacy of the final sample for prevalence estimation and multivariable modeling. Assuming an expected peri‐implantitis prevalence of 35% based on previous literature [19], with a two‐sided alpha of 0.05% and 80% power, the minimum required sample size was calculated to be 93 patients. To estimate implant‐level prevalence while accounting for clustering of implants within patients, the sample size was further adjusted using a design effect derived from an ICC of 0.3, yielding an adjusted minimum requirement of approximately 145 implants.

Given the anticipated nonresponse rate due to factors such as patient relocation, mortality, or lack of interest, it was estimated that contacting around 300 patients would likely result in a sufficiently powered final sample. This projection was consistent with the size of the existing patient registry, which included 397 individuals. Importantly, these calculations were performed retrospectively to contextualize the robustness of the final sample and not to guide recruitment.

### 2.5 Data Analysis

Statistical analyses were performed using STATA version 18 software (StataCorp LP). Descriptive characteristics were summarized at both patient and implant levels. The prevalence of peri‐implant health and disease was calculated, as well as the prevalence of PISTD. Implant survival rate was calculated descriptively, with a corresponding 95% confidence interval (CI). To evaluate the risk and protective indicators associated with peri‐implantitis, multilevel and multivariable logistic regression analyses were executed. This analysis focused on assessments of moderate/severe peri‐implantitis based on a mixed criterion, where direct evidence of bone loss was prioritized over the indirect criterion. Each potential indicator was assessed separately by incorporating it into an empty model, with moderate/severe peri‐implantitis status (yes or no) as the dependent variable, and evaluating its significance. Variables that were significant at the 0.10 level were included in an intermediate model, while nonsignificant variables were systematically excluded using a stepwise backward selection process. The final model comprised all factors that remained statistically significant (*p* < 0.05). To account for the nonindependence of implants within patients, multilevel logistic regression models were used with a random intercept at the patient level. Patient‐level variables (e.g., smoking status, periodontal diagnosis, and SPIC frequency) and implant‐level variables (e.g., implant position, soft‐tissue phenotype, and prosthetic design) were included in the model accordingly. The same strategy was employed for PISTD. Sensitivity analyses were conducted for moderate–severe peri‐implantitis (only indirect evidence) as well as for peri‐implantitis according to the 2017 World Workshop definition, as well as for PISTD only in implants without peri‐implantitis.

## 3 Results

A total of 397 patients met the inclusion criteria and a telephone contact was attempted. Of these, 251 were excluded due to the following reasons: unavailable or inactive contact numbers (134 patients, 36%), deceased or bedridden status (51 patients, 13%), lack of interest due to ongoing care by external providers (37 patients, 9%), and relocation abroad (20 patients, 5%).

As a result, 146 patients, with a total of 529 initially placed implants, participated in the present study. Retrospective records revealed that 18 implants had been lost in 11 of these patients—17 due to peri‐implantitis and one due to implant fracture—yielding an overall implant survival rate of 96.5% (95% CI: 94.9%–98.1%). Consequently, the final analyzed sample included 511 implants from 146 patients.

### 3.1 Characteristics of the Study Patients and Implants

Descriptive characteristics of the study population and implants are summarized in Table 1. The mean age of participants was 61.1 years (SD = 14.5; range 2–31 years), with a predominance of females (65.7%). Regarding smoking status, 50.0% were nonsmokers, 15.7% were former smokers, and 34.3% were current smokers. Periodontitis was highly prevalent, with 56.8% of patients having stage III and 8.3% having stage IV disease. Most of the included patients (53.4%) were under regular SPIC ≥ twice a year, while 15.1% attended irregularly or not at all.

**TABLE 1:** General characteristics of the study population and implants recorded at follow‐up.

Patient level	*N* = 146
Age (years), mean (SD)	61.1 (14.5)
Gender, *N* (%)	
Male	50 (34.3)
Female	96 (65.7)
Smoking status, *N* (%)	
Nonsmokers	73 (50.0)
Former smokers	23 (15.7)
Current smokers	50 (34.3)
Periodontal status (2017 WWP‐stage), *N* (%)	
No periodontitis	33 (22.6)
Stages I–II periodontitis	13 (8.9)
Stage III periodontitis	83 (56.8)
Stage IV periodontitis	12 (8.3)
Edentulous	5 (3.4)
Full mouth scores (excluding study implants), mean (SD)	
FMBS	34 (26.8)
FMPS	93 (73.2)
Regular SPIC attendance, *N* (%)	
No/sporadic	22 (15.1)
Once a year	46 (31.5)
Twice a year	70 (47.9)
> twice a year	8 (5.5)
Implant level	*N* = 511
Jaw, *N* (%)	
Maxilla	302 (59.1)
Mandible	209 (40.9)
Position, *N* (%)	
Anterior (canine‐canine)	115 (22.5)
Posterior	396 (77.5)
Implant brand, *N* (%)	
Nobel Biocare	467 (91.4)
Biomet 3i	33 (6.5)
Others	11 (2.1)
Type of prosthetic restoration, *N* (%)	
Single crown	215 (42.1)
Bridge	225 (44.0)
Full‐arch fixed restoration	64 (12.5)
Overdenture	7 (1.4)
Prosthesis retention, *N* (%)	
Cemented	207 (40.5)
Screw‐retained	304 (59.5)

Abbreviations: FMBS, full‐mouth bleeding score; FMPS, full‐mouth plaque score; N, number; SD, standard deviation; SPIC, supportive peri‐implant care; WWP, World Workshop in Periodontology.

Patients had a mean of 3.7 ± 2.6 implants (range 1–12), with a mean function time of 13.3 years (SD = 7.1; range 2–31 years). At the implant level, 59.1% were located in the maxilla, and 77.5% were placed in posterior regions. The most common prosthetic restorations were bridges (44.0%) and single crowns (42.1%). The predominant implant brand was Nobel Biocare (91.4%), followed by Biomet 3i (6.5%). No missing data were present for all the variables analyzed.

### 3.2 Prevalence of Peri‐Implant Health and Diseases

**FIGURE 2:**
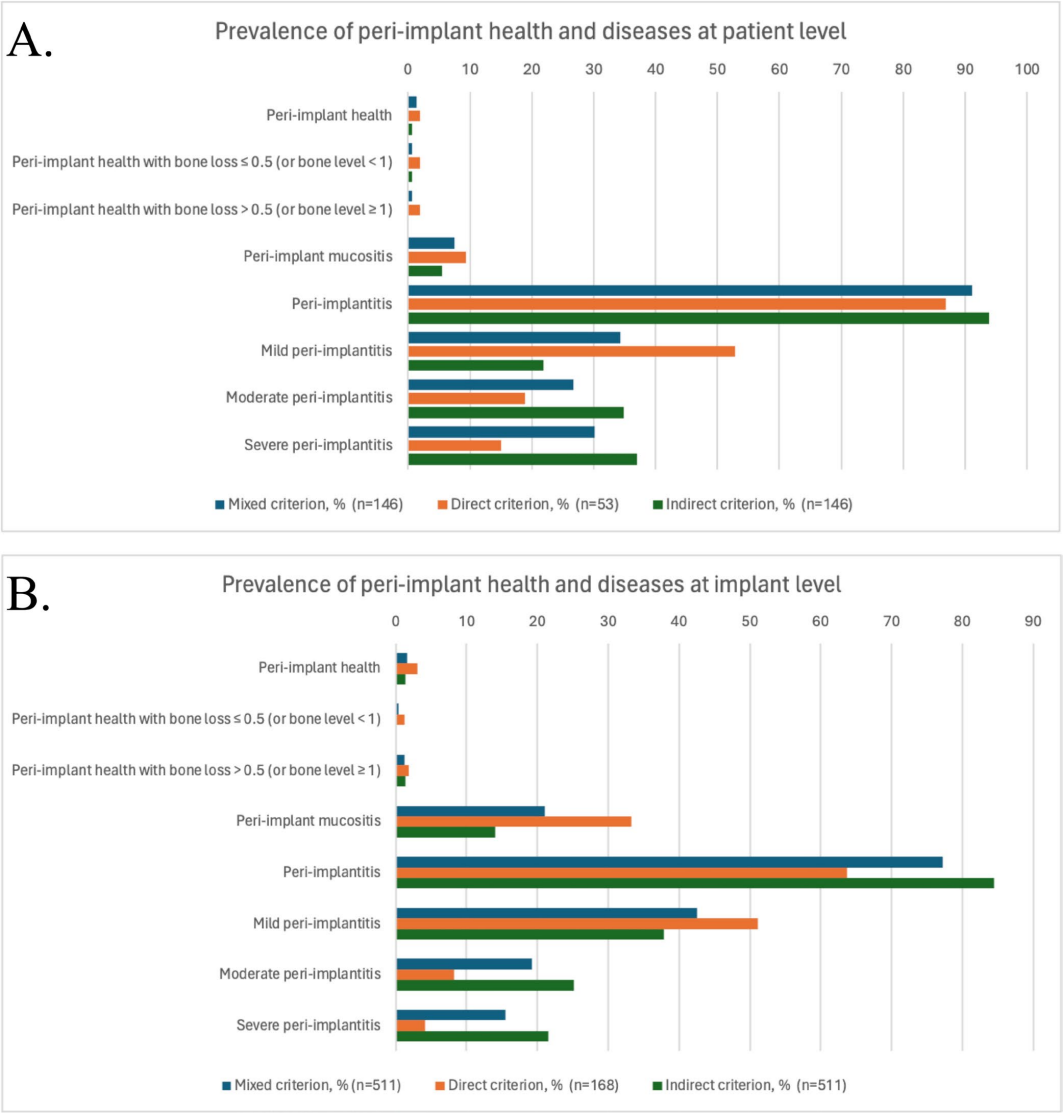
Bar graphs illustrating the prevalence of peri‐implantitis across diagnostic definitions at patient (A) and implant (B) levels. Mild peri‐implantitis was defined as bone loss ≥ 0.5 mm and < 2 mm (or bone level ≥ 1 mm and < 2 mm), moderate as ≥ 2 mm and < 3 mm, and severe as ≥ 3 mm, based on direct or indirect evidence.

The prevalence of peri‐implant health and disease, based on mixed, direct, and indirect diagnostic criteria, is reported in Table 2 and Figure 2. Reliable baseline radiographs were available for 168 out of 511 implants (32.9%). Table S1 presents the same data using an alternative inflammation threshold (≥ 4 BoP‐positive sites).

**TABLE 2:** Prevalence of peri‐implant health and diseases (Sanz 2012).

	Patient level			Implant level		
	Mixed criterion (*n* = 146)	Direct criterion (*n* = 53)	Indirect criterion (*n* = 146)	Mixed criterion (*n* = 511)	Direct criterion (*n* = 168)	Indirect criterion (*n* = 511)
Peri‐implant health, *N* (%)	2 (1.4)	2 (3.8)	1 (0.7)	8 (1.6)	5 (3.0)	7 (1.4)
With bone loss ≤ 0.5 (or bone level < 1)	1 (0.7)	1 (1.9)	1 (0.7)	2 (0.4)	2 (1.2)	0 (0)
With bone loss > 0.5 (or bone level ≥ 1)	1 (0.7)	1 (1.9)	0	6 (1.2)	3 (1.8)	7 (1.4)
Peri‐implant mucositis,^a^ *N* (%)	11 (7.5)	5 (9.4)	8 (5.5)	108 (21.1)	56 (33.3)	72 (14.1)
Peri‐implantitis, *N* (%)	133 (91.1)	46 (86.8)	137 (93.8)	395 (77.3)	107 (63.7)	432 (84.5)
Mild, *N* (%)	50 (34.3)	28 (52.8)	32 (21.9)	218 (42.6)	86 (51.2)	193 (37.8)
Moderate, *N* (%)	39 (26.7)	10 (18.9)	51 (34.9)	98 (19.2)	14 (8.3)	129 (25.2)
Severe, *N* (%)	44 (30.1)	8 (15.1)	54 (37.0)	79 (15.5)	7 (4.2)	110 (21.5)

*Note:* Direct criteria. Mild peri‐implantitis/pre‐peri‐implantitis: BoP + bone loss > 0.5 mm; Moderate peri‐implantitis: BoP + Bone loss ≥ 2 and < 3 mm; and Severe peri‐implantitis: BoP + Bone loss ≥ 3 mm. Indirect criteria. Mild peri‐implantitis/pre‐peri‐implantitis: BoP + bone level ≥ 1 and < 2 mm; Moderate peri‐implantitis: BoP + Bone level ≥ 2 and < 3 mm; and Severe peri‐implantitis: BoP + Bone level ≥ 3 mm. Mixed criteria. Direct evidence or indirect evidence for implants without previous radiographs.

Abbreviations: BoP, bleeding on probing; *N*, number.

^a^ Patients with mucositis without peri‐implantitis.

Using the mixed diagnostic criterion, 91.1% of patients were diagnosed with peri‐implantitis, while peri‐implant health was observed in only 1.4%. At the patient level, the distribution of peri‐implantitis severity was 34.3% mild, 26.7% moderate, and 30.1% severe. At the implant level, peri‐implantitis affected 77.3% of implants, with 42.6% classified as mild, 19.2% as moderate, and 15.5% as severe. Peri‐implant health was present in just 1.6% of implants.

When applying the direct criterion only, peri‐implantitis prevalence at the patient level increased to 52.8% (mild), while moderate and severe forms decreased to 18.9% and 15.1%, respectively. Peri‐implant health increased to 3.8%. At the implant level, prevalence of peri‐implantitis rose to 51.2% (mild), while moderate and severe forms dropped to 8.3% and 4.2%, respectively. Peri‐implant health increased to 3.0%.

Conversely, when using the indirect criterion alone, peri‐implantitis prevalence at the patient level decreased to 21.9% (mild), while moderate and severe cases increased to 34.9% and 37.0%, respectively. Peri‐implant health was observed in only 0.7% of patients. At the implant level, peri‐implantitis prevalence slightly decreased to 37.8% (mild), while moderate and severe cases increased to 25.2% and 21.5%, respectively. Peri‐implant health was identified in 1.4% of implants. The differences comparing the direct and indirect criteria both at the patient and implant level were statistically significant (*p* < 0.001).

A sensitivity analysis applying the indirect criterion proposed by the 2017 World Workshop on Periodontal and Peri‐Implant Diseases and Conditions yielded a significant decrease in peri‐implantitis prevalence to 24.7% at the patient level and to 14.3% at the implant level. Corresponding peri‐implant mucositis prevalence was 62.3% and 79.8%, respectively (see Appendix A (Tables A1, A2, A3)). The differences in prevalence estimates obtained using the Sanz and Chapple [18] versus Berglundh et al. [1] case definitions—based on indirect criteria and applied to the same individuals—were also statistically significant (*p* < 0.001).

### 3.3 Prevalence of Buccal PISTD

The prevalence of buccal PISTD is summarized in Table 3. The majority of PISTD were located in the posterior region (73.9%) and in the maxilla (60.9%).

**TABLE 3:** Prevalence of buccal peri‐implant soft‐tissue dehiscences.

	Patient level			Implant level		
	Overall PISTD	PISTD with PI^a^	PISTD without PI^a^	Overall PISTD	PISTD with PI^a^	PISTD without PI^a^
Absence of PISTD, *N* (%)	67 (45.9)	42 (40.0)	25 (61.0)	304 (59.5)	125 (52.3)	179 (65.8)
Presence of PISTD, *N* (%)						
Total (> 0 mm)	79 (54.1)	63 (60.0)	16 (39.0)	207 (40.5)	114 (47.7)	93 (34.2)
> 1 mm	35 (24.0)	32 (30.5)	3 (8.6)	73 (14.3)	46 (19.2)	27 (9.9)
> 2 mm	10 (6.8)	8 (7.6)	2 (4.9)	22 (4.3)	11 (4.6)	11 (4.0)

Abbreviations: *N*, number; PI, peri‐implantitis; PISTD, peri‐implant soft‐tissue dehiscence.

^a^ Corresponding to the Sanz and Chapple (2012) peri‐implantitis case definition.

At patient level, 54.1% had at least one implant with buccal PISTD (> 0 mm). Among patients with peri‐implantitis, this figure rose to 60.0%, compared to 39.0% in those without peri‐implantitis (*p* = 0.027). At implant level, buccal PISTD affected 40.5% of all implants, with higher prevalence in implants with peri‐implantitis (47.7%) than in those without (34.2%; *p* = 0.002). PISTD > 1 mm was found in 24.0% of patients and 14.3% of implants. PISTD > 2 mm affected 6.8% of patients and 4.3% of implants.

### 3.4 Risk Indicators of Moderate/Severe Peri‐Implantitis

The distribution of potential risk and protective indicators according to moderate/severe peri‐implantitis and the corresponding univariable analyses are provided in Tables S2 and S3. The final multilevel logistic regression model identified several risk indicators of moderate/severe peri‐implantitis (Table 4): stage III–IV periodontitis (OR = 2.82; 95% CI: 1.30–6.15), loading time ≥ 10 years (OR = 3.02; 95% CI: 1.55–5.89), bisphosphonate use (OR = 5.96; 95% CI: 1.33–26.66), and presence of a cantilever (OR = 5.51; 95% CI: 1.56–19.38). Conversely, the following emerged as protective indicators: regular SPIC (> twice/year) (OR = 0.16; 95% CI: 0.03–0.95), presence of > 2 mm of keratinized tissue height (OR = 0.44; 95% CI: 0.21–0.95), and correct mesio‐distal implant positioning (OR = 0.54; 95% CI: 0.32–0.94).

**TABLE 4:** Risk and protective indicators associated with moderate/severe peri‐implantitis (mixed case definition).

Variables	Empty model		Final model		
	OR	95% CI	OR	95% CI	*p*‐value
Fixed part					
Intercept	0.40	0.28–0.56	0.36	0.10–1.32	
Regular SPIC attendance					
No/sporadic			Ref		
Once a year			0.48	0.18–1.25	0.132
Twice a year			0.52	0.22–1.24	0.138
> twice a year			0.16	0.03–0.95	0.044
Periodontal diagnosis^a^					
No periodontitis			Ref		
Stages I–II			2.21	0.68–7.16	0.186
Stages III–IV			2.82	1.30–6.15	0.009
Bisphosphonates					
No			Ref		
Yes			5.96	1.33–26.66	0.020
Cantilever					
No			Ref		
Yes			5.51	1.56–19.38	0.008
Function time					
< 10 years			Ref		
≥ 10 years			3.02	1.55–5.89	0.001
Keratinized tissue height (buccal)			Ref		
0 mm			0.52	0.24–1.12	0.093
1–2 mm			0.44	0.21–0.95	0.038
> 2 mm					
Mesio‐distal positioning					
Incorrect			Ref		
Correct			0.54	0.32–0.94	0.031
Random part					
Patient variance	1.90	1.01–3.59	1.17	0.53–2.61	
AIC	597.702		616.563		

*Note:* A likelihood‐ratio test comparing the model to ordinary logistic regression was performed and identified as highly significant for these data (*p* < 0.001). The intra‐class correlation (ICC) at the patient level showed that 36.6% (ICC 0.36; 95% CI: 0.23–0.52) of the correlation was due to variation among patients and 63.4% due to variations among implants.

Abbreviations: AIC, Akaike's information criterion; CI, confidence interval; OR, odds ratio; Ref, reference category; SPIC, supportive peri‐implant care.

^a^ Periodontal status refers to the examination visit. Edentulous were considered either to have no periodontitis or stage IV periodontitis based on the reason for tooth loss.

Sensitivity analyses using the indirect diagnostic criteria for peri‐implantitis proposed by the 2012 and 2017 World Workshops yielded largely consistent results (Appendix A). Among the differential findings, access to interproximal oral hygiene emerged as a protective factor in the 2012 definition (OR = 0.46; 95% CI: 0.21–0.98), whereas mesio‐distal positioning was not significantly associated. Additionally, tooth loss due to trauma was associated with increased risk under the 2017 classification (OR = 5.02; 95% CI: 1.31–19.21), and keratinized tissue height of 1–2 mm remained a protective factor (OR = 0.08; 95% CI: 0.03–0.23).

### 3.5 Risk Indicators of Buccal PISTD

The final multilevel logistic regression model identified several risk indicators of buccal PISTD (Table 5): diagnosis of peri‐implantitis (OR = 2.21; 95% CI: 1.25–3.91), use of intermediate abutments (OR = 4.92; 95% CI: 1.92–12.58), and position adjacent to another implant (OR = 3.35; 95% CI: 1.50–7.48) or to an edentulous space (OR = 3.38; 95% CI: 1.51–7.54). In contrast, protective indicators for PISTD included the following: mandibular location (OR = 0.45; 95% CI: 0.25–0.81), thick buccal soft‐tissue phenotype (OR = 0.18; 95% CI: 0.08–0.42), and buccal keratinized tissue height of 1–2 mm (OR = 0.16; 95% CI: 0.06–0.46) or > 2 mm (OR = 0.05; 95% CI: 0.02–0.15).

**TABLE 5:** Risk/protective indicators associated with the presence of PISTD.

Variables	Empty model		Final model		
	OR	95% CI	OR	95% CI	*p*‐value
Intercept					
Fixed part	0.43	0.29–0.63	1.76	0.35–8.90	
Jaw					
Maxilla			Ref		
Mandible			0.45	0.25–0.81	0.007
Buccal phenotype					
Thin			Ref		
Thick			0.18	0.08–0.42	< 0.001
Intermediate abutment					
No			Ref		
Yes			4.92	1.92–12.58	0.001
Adjacent element					
Tooth			Ref		
Implant			3.35	1.50–7.48	0.003
Edentulous			3.38	1.51–7.54	0.003
Peri‐implantitis					
No			Ref		
Yes			2.21	1.25–3.91	0.007
Keratinized tissue height (buccal)					
0 mm			Ref		
1–2 mm			0.16	0.06–0.46	0.001
> 2 mm			0.05	0.02–0.15	< 0.001
Random part					
Patient variance	2.29	1.26–4.17	1.79	0.83–3.86	
AIC	523.518		633.099		

*Note:* A likelihood‐ratio test comparing the model to ordinary logistic regression was performed and identified as highly significant for these data (*p* < 0.001). The intra‐class correlation (ICC) at the patient level showed that 35.3% (ICC 0.35; 95% CI 0.20–0.54) of the correlation was due to variation among patients and 64.7% due to variations among implants. Moderate–severe peri‐implantitis was considered according to Sanz and Chapple (2012).

Abbreviations: AIC, Akaike's information criterion; CI, confidence interval; N, number; OR, odds ratio; PISTD, peri‐implant soft‐tissue dehiscence; Ref, reference category.

The results of the sensitivity analysis conducted on implants not affected by peri‐implantitis are reported in the Appendix A. Findings were consistent with the main analysis, with the addition of a protective association for correct bucco‐lingual implant positioning (OR = 0.32; 95% CI: 0.10–0.98) and a strong positive association between bisphosphonate use and the presence of PISTD (OR = 32.20; 95% CI: 2.35–440.51).

## 4 Discussion

In this cross‐sectional study conducted within a long‐term university‐based cohort, peri‐implantitis and buccal PISTD emerged as highly prevalent complications, despite overall high implant survival. Multilevel logistic regression identified several key risk indicators for peri‐implantitis, including advanced stages of periodontitis, extended implant function time, use of bisphosphonates, and the presence of cantilevered restorations. In contrast, regular supportive care, the presence of adequate keratinized mucosa, and proper mesio‐distal implant positioning appeared to have a protective effect. Regarding PISTD, its occurrence was more likely in association with peri‐implantitis, with the presence of intermediate abutments, and in implants adjacent to other implants or to edentulous areas. Protective factors included mandibular location, a thick buccal soft‐tissue phenotype, and sufficient buccal keratinized tissue. Sensitivity analyses applying different case definitions revealed some variability in prevalence estimates, but the overall pattern of risk and protective indicators remained consistent.

Compared to previous systematic reviews reporting implant‐level peri‐implantitis prevalence of 21.7%–albeit with high heterogeneity [3], this study identified substantially higher rates. Specifically, the cumulative peri‐implantitis prevalence at the patient level using the mixed criterion reached 91.1%, which is indeed striking and warrants careful interpretation. This elevated estimate likely reflects multiple factors: first, the case definition adopted was inclusive, combining both direct and indirect diagnostic criteria, which may have increased diagnostic sensitivity, particularly in cases without baseline radiographs (67.1% of cases). In support of this, when only direct evidence was applied, the estimated prevalence was significantly lower. Similarly, when applying the 2017 World Workshop case definition, which uses a threshold of ≥ 3 mm bone level and PPD ≥ 6 mm, prevalence was also substantially reduced. Second, plaque control in this cohort was poor, potentially contributing to the high peri‐implant diseases estimates exceeding prior reports [3]. This may relate to age‐related limitations in oral hygiene and the fact that nearly half of patients did not attend regular supportive care or were followed at generalist dental clinics. Although with some differences, these patterns align with the higher disease prevalence observed in southern European populations relative to other cohorts [4, 5, 19]. Furthermore, the longer mean implant function time in this study (13.3 years) likely contributed to the elevated prevalence, as most prior studies included implants with shorter durations in function [4, 17, 19].

Despite the high peri‐implantitis prevalence, long‐term implant survival remained high and consistent with the available literature [30, 31, 32, 33]. This apparent paradox—where peri‐implantitis is widespread yet many implants remain functional—was already epitomized by the diverging meaning of survival versus success [34], but it may also raise questions about the clinical relevance of current diagnostic thresholds [35]. Not all peri‐implantitis cases may be at equal risk of progression, and existing criteria may fail to distinguish between stable and progressive forms of the disease. Given the cross‐sectional design, disease progression could not be assessed [16]. However, caution is warranted in interpreting the high implant survival rates. While implant loss was systematically assessed and no respondent explicitly declined participation due to complete implant failure, it remains possible that individuals who had lost all implants were less likely to be reachable or willing to participate. This potential nonresponse bias could lead to a slight overestimation of implant survival and underscores the importance of considering patient attrition when evaluating long‐term outcomes. Future longitudinal studies are needed to distinguish between stable and progressive lesions and to develop predictive diagnostic models, potentially integrating biomarkers for active disease.

The findings from this study also challenge the interpretation of BoP as a sole diagnostic marker around implants. Nearly all implants (99%) exhibited at least one BoP‐positive site, and 96% had two or more, raising the concern that BoP—while a valid indicator of soft tissue inflammation—may not reliably reflect disease activity or ongoing tissue breakdown in the peri‐implant context [36, 37]. This does not imply that BoP should be disregarded, but rather that its diagnostic specificity may be limited in populations with poor plaque control and high baseline inflammation. The mere presence of bleeding may therefore contribute to overestimation of disease prevalence and potentially underestimate the true efficacy of treatment procedures if not interpreted alongside radiographic and longitudinal clinical parameters [38, 39, 40]. Indeed, around implants, BoP can be influenced by several factors—including the depth of the transmucosal tunnel, prosthesis morphology, and probing accessibility—which may elicit bleeding responses even in the absence of significant peri‐implant pathology [41].

Multilevel regression analyses identified several risk and protective indicators for moderate/severe peri‐implantitis. Stage III–IV periodontitis was strongly associated with peri‐implantitis, supporting prior findings [5, 19, 42, 43, 44, 45]. Potential mechanisms include persistent microbial reservoirs [46, 47], and dysregulated background immune responses [17, 48]. Implant function time ≥ 10 years emerged as a significant risk indicator for moderate‐to‐severe peri‐implantitis, reinforcing the association between long‐term function and bone loss, potentially due to the cumulative effect of other risk factors over time [3, 49]. Prosthetic design and biomechanical aspects also played a role. Cantilever prostheses were associated with moderate/severe peri‐implantitis, likely due to impaired hygiene access or unfavorable biomechanics. The use of bisphosphonates was also associated with increased risk, although only six patients in the cohort were receiving this medication. This may suggest a potential role of altered bone metabolism in compromising peri‐implant hard tissue stability [50]. Regular SPIC was a protective indicator, consistent with studies demonstrating lower peri‐implantitis risk among SPIC attendees [12, 20, 48, 51]. Adequate keratinized mucosa (≥ 2 mm) was protective, supporting its role in enhancing hygiene comfort and providing a mechanical seal of the peri‐implant mucosa [20, 52]. Sensitivity analyses additionally identified adequate interproximal space for hygiene access as a protective indicator, in line with previous findings [4, 19]. Conversely, tooth loss due to trauma was associated with a higher risk of peri‐implantitis, potentially reflecting more complex anatomical conditions that may increase the likelihood of implant malposition or surgical complications. The lack of detailed surgical records in most clinical charts, along with the absence of 3D bone volume assessments at the time of implant placement, represents a potential source of information bias in this context. Several risk factors previously reported as risk indicators for peri‐implantitis–such as smoking [5], diabetes [53], implant brand/surfaces [5, 17, 54], type of restoration [4, 5], excessive initial bone remodeling [55], and bucco‐lingual implant misposition [5]–did not emerge as significant in the present study. Differences in population characteristics and limited statistical power due to testing for multiple variables may partially explain this discrepancy. Also, large ORs for bisphosphonate use and cantilever may reflect residual confounding or small subgroup effects, and should be interpreted cautiously. Notably, several of the identified indicators—such as prosthetic design (e.g., cantilevers), SPIC frequency, and soft‐tissue characteristics—are modifiable through clinical planning and maintenance protocols, highlighting potential targets for prevention and individualized risk reduction strategies. Furthermore, as periodontal status was assessed at the time of the examination, it may reflect a transient condition; this leaves room for risk modification over time if periodontitis is successfully treated and maintained under control.

The prevalence of buccal PISTD observed in the present study was higher than that reported in a previous university‐based sample focused on anterior implants, which may be explained by the inclusion of posterior sites and the longer implant function time in the current cohort [24]. Here, the presence of PISTD appeared to be associated with both anatomical and prosthetic factors, with buccal soft‐tissue phenotype, correct implant positioning, and the presence of peri‐implantitis showing a prominent effect. Importantly, the relationship between peri‐implant bone loss and buccal soft‐tissue dehiscence also carries potential esthetic and patient‐centered implications. PISTD, particularly in the anterior maxilla, can lead to compromised esthetic outcomes, visible metallic or ceramic exposure, and dissatisfaction with implant‐supported restorations—issues increasingly recognized as relevant to patients' quality of life and overall treatment success [24, 56]. A thick buccal soft‐tissue phenotype and the presence of keratinized tissue height emerged as protective indicators, consistent with previous reports [57, 58, 59, 60, 61]. Lack of keratinized tissue may also be a consequence, rather than a cause, of PISTD or peri‐implantitis—an aspect that cannot be resolved within the present cross‐sectional design [59, 62]. In this cohort, posterior implants were more frequently rehabilitated with intermediate abutments placed supra‐ or peri‐mucosally to enhance cleanability, increasing the likelihood of visible abutment exposure and potentially contributing to the higher PISTD prevalence in these regions. In fact, most dehiscences in our cohort were located in the posterior region (73.9%) and the maxilla (60.9%), a distribution that likely influenced the overall prevalence. However, since these sites are generally less critical from an esthetic standpoint, their clinical relevance may not necessarily translate into patient dissatisfaction. Among the other risk indicators, implants adjacent to another implant or to an edentulous space were more frequently affected by PISTD compared to those adjacent to natural teeth, suggesting that the absence of the periodontal attachment and the natural remodeling of the alveolar crest that may continue even long after tooth extraction can negatively influence tissue preservation [63]. As expected, the presence of peri‐implantitis was associated with PISTD, consistent with previous findings suggesting that peri‐implant bone loss may manifest clinically as soft‐tissue recession [24, 64, 65]. Also for PISTD, the large ORs and CI for bisphosphonate use strongly suggest a small subgroup effect and should be interpreted cautiously. Overall, the case definition of PISTD—specifically the inclusion of abutment exposure—may have influenced prevalence estimates. To improve comparability across future studies, the adoption of standardized definitions that clearly distinguish between clinically relevant mucosal recessions and prosthetic design‐related exposures is suggested, ideally integrating both dimensional thresholds and esthetic impact [66, 67].

Study limitations include the cross‐sectional design, which prevents causal inference [68]. The university‐based cohort may limit generalizability, and reliance on self‐reported data records for some parameters (SPIC frequency, previous treatments) poses the risk of information bias, despite cross‐checking with patient records. Additionally, while the predominance of a single implant brand (91.4%) ensured a relatively homogeneous sample in terms of implant macro‐ and micro‐design, this also limits generalizability to other systems. Prospective studies are needed to validate these findings and refine risk stratification, possibly based on biological phenotyping [69, 70, 71].

## 5 Conclusion

This study revealed a high prevalence of peri‐implantitis and buccal PISTD in a long‐term university‐based cohort, with disease rates significantly influenced by the diagnostic criteria applied. Despite the widespread occurrence of peri‐implant diseases, implant survival remained high, challenging the clinical relevance of current diagnostic thresholds and underscoring the need for refined, progression‐based definitions. Future longitudinal studies should focus on distinguishing between stable and progressive disease forms. Multiple patient‐ and implant‐level factors emerged as significant risk or protective indicators for peri‐implantitis and buccal PISTD, highlighting the implementation of individualized risk assessment and supportive peri‐implant care strategies to reduce peri‐implant complications.

## Author Contributions

G.B. contributed to conception and design, data acquisition, analysis and interpretation, drafted and critically revised the manuscript; F.R. contributed to data analysis and interpretation, drafted and critically revised the manuscript; S.C., M.C., A.L.G., M.V. contributed to clinical procedures, data acquisition, and drafted the manuscript; G.M.M. contributed to design, data acquisition and interpretation, drafted and critically revised the manuscript; M.R. contributed to conception and design, data analysis and interpretation, and critically revised the manuscript; G.S. contributed to clinical procedures, interpretation, and critically revisited the manuscript; M.A. contributed to conception and design, and critically revised the manuscript. All authors gave their final approval and agree to be accountable for all aspects of the work.

## Disclosure

AI Statement: The authors utilized ChatGPT (OpenAI) to assist with language refinement and final proofreading of the manuscript. All scientific content, interpretations, and conclusions are the sole responsibility of the authors.

## Conflicts of Interest

The authors declare no conflicts of interest.

## Supporting information


**Appendix S1:** jre70025‐sup‐0001‐AppendixS1.docx.

## Data Availability

The data of this study are available from the corresponding author upon reasonable request.

## Appendix A

**TABLE A1:** Sensitivity analysis for risk and protective indicators associated with moderate–severe peri‐implantitis using the Sanz and Chapple 2012 definition.

Variables	Empty model		Final model		
	OR	95% CI	OR	95% CI	*p*‐value
Fixed part					
Intercept	0.82	0.59–1.12	0.87	0.25–3.02	
Regular SPIC attendance					
No/sporadic			Ref		
Once a year			0.48	0.18–1.28	0.143
Twice a year			0.49	0.20–1.18	0.112
> twice a year			0.16	0.03–0.84	0.030
Periodontal diagnosis^a^					
No periodontitis			Ref		
Stages I–II			1.43	0.44–4.63	0.552
Stages III–IV			2.36	1.05–5.31	0.037
Cantilever					
No			Ref		
Yes			6.93	1.66–29.01	0.008
Function time					
< 10 years			Ref		
≥ 10 years			2.71	1.43–5.17	0.002
Keratinized tissue height (buccal)					
0 mm			Ref		
1–2 mm			0.37	0.17–0.83	0.016
> 2 mm			0.37	0.17–0.84	0.017
Access to oral hygiene					
No			Ref		
Yes			0.46	0.21–0.98	0.046
Implant‐abutment connection					
External			Ref		
Internal			1.35	0.77–2.37	0.295
Conical			3.07	1.21–7.79	0.018
Random part					
Patient variance	1.85	1.00–3.43	1.23	0.57–2.64	
AIC	643.025		663.096		

*Note:* A likelihood‐ratio test comparing the model to ordinary logistic regression was performed and identified as highly significant for these data (*p* < 0.001). The intra‐class correlation (ICC) at the patient level showed that 27.2% (ICC 0.27; 95% CI 0.15–0.44) of the correlation was due to variation among patients and 72.8% due to variations among implants.

Abbreviations: AIC, Akaike's information criterion; CI, confidence interval; OR, odds ratio; Ref, reference category; SPIC, supportive peri‐implant care.

^a^ Edentulous were considered either no periodontitis or stage IV periodontitis based on the reason for tooth loss.

**TABLE A2:** Sensitivity analysis for risk and protective indicators associated with moderate–severe peri‐implantitis using the WW17 case definition.

Variables	Empty model		Final model		
	OR	95% CI	OR	95% CI	*p*‐value
Fixed part					
Intercept	0.05	0.02–0.12	0.03	0.00–0.18	
Regular SPIC attendance					
No/sporadic			Ref		
Once a year			0.34	0.04–2.78	0.313
Twice a year			0.30	0.11–0.81	0.018
> twice a year			0.15	0.04–0.55	0.004
Periodontal diagnosis^a^					
No periodontitis			Ref		
Stages I–II			7.11	1.08–46.73	0.041
Stages III–IV			11.51	2.75–48.12	0.001
Bisphosphonates					
No			Ref		
Yes			7.21	1.33–39.20	0.022
Cantilever					
No			Ref		
Yes			8.52	1.89–38.39	0.005
Reason of tooth loss					
Caries			Ref		
Periodontitis			0.59	0.24–1.44	0.243
Trauma, fracture			5.02	1.31–19.21	0.018
Agenesia			0.70	0.06–8.64	0.783
Function time					
< 10 years			Ref		
≥ 10 years			6.56	2.22–19.36	0.001
Keratinized tissue height (buccal)					
0 mm			Ref		
1–2 mm			0.08	0.03–0.23	< 0.001
> 2 mm			0.38	0.16–0.90	0.028
Random part					
Patient variance	3.59	1.58–8.15	0.70	0.14–3.54	
AIC	380.471		333.482		

*Note:* A likelihood‐ratio test comparing the model to ordinary logistic regression was performed and identified as highly significant for these data (*p* < 0.001). The intra‐class correlation (ICC) at the patient level showed that 17.6% (ICC 0.18; 95% CI 0.04–0.52) of the correlation was due to variation among patients and 81.4% due to variations among implants.

Abbreviations: AIC, Akaike's information criterion; CI, confidence interval; OR, odds ratio; Ref, reference category; SPIC, supportive peri‐implant care.

^a^ Edentulous were considered either no periodontitis or stage IV periodontitis based on the reason for tooth loss.

**TABLE A3:** Risk/protective indicators associated with the presence of PISTD in implants without peri‐implantitis.

Variables	Empty model		Final model		
	OR	95% CI	OR	95% CI	*p*‐value
Fixed part					
Intercept	0.28	0.15–0.51	4.22	0.14–124.94	
Gender					
Male			Ref		
Female			2.53	0.99–6.48	0.053
Buccal phenotype					
Thin			Ref		
Thick			0.20	0.07–0.58	0.003
Bisphosphonates					
No			Ref		
Yes			32.20	2.35–440.51	0.009
Intermediate abutment					
No			Ref		
Yes			17.03	2.62–110.49	0.003
Correct placement (bucco‐lingual)					
No			Ref		
Yes			0.32	0.10–0.98	0.047
Keratinized tissue height (buccal)					
0 mm			Ref		
0–2 mm			0.05	0.00–0.56	0.015
> 2 mm			0.02	0.00–0.22	0.001
Random part					
Patient variance	3.52	1.44–7.82	1.03	0.22–4.88	
AIC	254.385		256.322		

*Note:* A likelihood‐ratio test comparing the model to ordinary logistic regression was performed and identified as highly significant for these data (*p* < 0.001). The intra‐class correlation (ICC) at the patient level showed that 23.8% (ICC 0.24; 95% CI 0.06–0.59) of the correlation was due to variation among patients and 64.7% due to variations among implants.

Abbreviations: AIC, Akaike's information criterion; CI, confidence interval; OR, odds ratio; Ref, reference category.
