# Supplementary material for: Prevalence and Risk Indicators of Peri‐Implant Diseases and Buccal Soft‐Tissue Dehiscence: A Cross‐Sectional Study From a University‐Based Cohort
Source: J Periodontal Res. 2025 Aug 7;61(3):256–72. doi: 10.1111/jre.70025 (PMC13140783; doi:10.1111/jre.70025)
Supplement: Supplementary file 1 — Appendix S1: jre70025‐sup‐0001‐AppendixS1.docx. [file JRE-61-256-s001.docx]

*Table S1. Prevalence of peri-implant health and diseases (inflammation cut-off BoP ≥ 4)*

|  | Patient level | | | Implant level | | |
| --- | --- | --- | --- | --- | --- | --- |
|  | Mixed criterion (n=146) | Direct criterion (n=53) | Indirect criterion  (n=146) | Mixed criterion (n=511) | Direct criterion (n=168) | Indirect criterion  (n=511) |
| Peri-implant health, N (%) | 19 (13.0) | 7 (8.3) | 18 (12.3) | 111 (21.7) | 36 (21.4) | 110 (21.5) |
| With bone loss ≤ 0.5 (or bone level < 1) | 4 (2.7) | 1 (1.2) | 4 (2.7) | 31 (6.1) | 15 (8.9) | 16 (0.3) |
| With bone loss > 0.5 (or bone level ≥ 1) | 15 (10.3) | 6 (7.1) | 14 (9.6) | 80 (15.6) | 21 (11.5) | 94 (18.4) |
| Peri-implant mucositis, N (%) | 13 (8.9) | 5 (9.1) | 9 (6.2) | 83 (16.2) | 43 (25.6) | 56 (11.0) |
| Peri-implantitis, N (%) | 114 (78.1) | 43 (78.2) | 119 (81.5) | 317 (62.1) | 89 (53.0) | 345 (67.5) |
| Mild, N (%) | 44 (30.1) | 27 (49.1) | 26 (17.8) | 170 (33.3) | 70 (41.7) | 149 (29.2) |
| Moderate, N (%) | 30 (20.6) | 9 (16.4) | 42 (28.8) | 75 (14.7) | 12 (7.1) | 97 (19.0) |
| Severe, N (%) | 40 (27.4) | 7 (12.7) | 51 (34.9) | 72 (14.1) | 7 (4.2) | 99 (19.4) |

*Table S2. Distribution of the putative patient-level risk/protective indicators in the study population, overall and according to peri-implantitis status and univariate models for moderate-severe peri-implantitis (mixed criterion).*

| **Variable** | **Overall (N=146)** | **Peri-implantitis status** | | **OR** | **95% CI** | **P value** |
| --- | --- | --- | --- | --- | --- | --- |
|  |  | **No peri-implantitis** | **Peri-implantitis** |  |  |  |
| Age (years), mean (SD) | 61.1 (14.5) | 56.7 (15.2) | 64.4 (13.0) | 1.02 | 0.99-1.04 | 0.137 |
| Gender, N (%)  Male  Female | 50 (34.3)  96 (65.7) | 23 (36.5)  40 (63.5) | 27 (32.5)  56 (67.5) | *Ref*  0.99 | 0.50-2.00 | 0.995 |
| BMI (kg/m2), mean (SD) | 24.3 (3.8) | 24.1 (3.2) | 24.5 (4.1) | 0.94 | 0.87-1.03 | 0.219 |
| Smoking status, N (%)  Non-smokers  Former smokers  Current smokers | 73 (50.0)  23 (15.7)  50 (34.3) | 33 (52.4)  8 (12.7)  22 (34.9) | 40 (48.2)  15 (18.1)  28 (33.7) | *Ref*  2.60  1.42 | 0.97-6.97  0.70-2.87 | 0.058  0.331 |
| Alcohol consumption status, N (%)  Never  Less than 2/week  Almost every day  Every day | 56 (38.4)  50 (34.2)  28 (19.2)  12 (8.2) | 21 (33.3)  22 (34.9)  15 (23.8)  5 (7.9) | 35 (42.2)  28 (33.7)  13 (15.7)  7 (8.4) | *Ref*  0.86  0.52  0.71 | 0.39-1.87  0.20-1.35  0.21-2.42 | 0.708  0.184  0.584 |
| Hypertension status, N (%)  No hypertension  Hypertension | 97 (66.4)  49 (33.6) | 45 (71.4)  18 (28.6) | 52 (62.6)  31 (37.4) | *Ref*  0.80 | 0.41-1.59 | 0.527 |
| Diabetes status, N (%)  No diabetes  Diabetes type 1  Diabetes type 2 | 138 (94.5)  5 (3.4)  3 (2.1) | 61 (96.8)  2 (3.2)  0 (0.0) | 77 (92.8)  3 (3.6)  3 (3.6) | *Ref*  0.77  0.69 | 0.13-4.42  0.08-5.93 | 0.772  0.739 |
| Osteoporosis, N (%)  No  Yes | 133 (91.1)  13 (8.9) | 60 (95.2)  3 (4.8) | 73 (88.0)  10 (12.0) | *Ref*  1.80 | 0.62-5.22 | 0.277 |
| Current medications, N (%)  No  Yes | 52 (35.6)  94 (64.4) | 23 (36.5)  40 (63.5) | 29 (34.9)  54 (65.1) | *Ref*  0.80 | 0.39-1.61 | 0.528 |
| Bisphosphonates, N (%)  No  Yes | 140 (95.9)  6 (4.1) | 62 (98.4)  1 (1.6) | 78 (94.0)  5 (6.0) | *Ref*  *3.93* | 0.81-19.10 | 0.089 |
| Proton pump inhibitors, N (%)  No  Yes | 125 (85.6)  21 (14.4) | 57 (90.5)  6 (9.5) | 68 (81.9)  15 (18.1) | *Ref*  1.23 | 0.52-2.94 | 0.635 |
| Vitamin D supplements, N (%)  No  Yes | 113 (77.3)  33 (14.4) | 49 (77.8)  14 (22.2) | 64 (77.1)  19 (22.9) | *Ref*  0.88 | 0.40-1.92 | 0.745 |
| Antidepressant/anxiolytic/antipsychotics, N (%)  No  Yes | 133 (91.1)  13 (8.9) | 56 (88.9)  7 (11.1) | 77 (92.8)  6 (7.2) | *Ref*  1.19 | 0.38-3.73 | 0.769 |
| Periodontal status (2017 WWP-stage), N (%)  No periodontitis  Stage I-II periodontitis  Stage III-IV periodontitis  Edentulous | 33 (22.6)  13 (8.9)  95 (65.1)  5 (3.4) | 17 (27.0)  7 (11.1)  39 (61.9)  0 (0.0) | 16 (19.3)  6 (7.2)  56 (67.5)  5 (6.0) | *Ref*  1.50  2.54  6.94 | 0.40-5.58  1.12-5.79  1.28-37.62 | 0.547  0.026  0.025 |
| Full mouth scores (excluding study implants), mean (SD)  FMBS  FMPS | 34 (26.8)  93 (73.2) | 50.4 (19.9)  68.4 (22.4) | 55.0 (24.6)  69.6 (22.2) | 1.01  1.01 | 1.00-1.03  0.99-1.02 | 0.037  0.889 |
| Regular SPIC attendance, N (%)  No/sporadic  Once a year  Twice a year  > twice a year | 22 (15.1)  46 (31.5)  70 (48.0)  8 (5.5) | 7 (11.1)  20 (31.7)  30 (47.6)  6 (9.5) | 15 (18.1)  26 (31.3)  40 (48.2)  2 (2.4) | *Ref*  0.61  0.62  0.14 | 0.23-1.67  0.24-1.57  0.02-0.97 | 0.341  0.313  0.046 |
| Previous periodontal treatment, N (%)  No  Yes | 89 (61.0)  57 (39.0) | 38 (60.3)  25 (39.7) | 51 (61.4)  32 (38.6) | *Ref*  1.27 | 0.65-2.50 | 0.486 |

*Abbreviation: BMI, bone mass index; FMBS, full-mouth bleeding score; FMPS, full-mouth plaque score; N; number; SD, standard deviation; SPIC, supportive peri-implant care; WWP, World Workshop in Periodontology*

*Table S3. Distribution of the putative implant-level risk/protective indicators in the study population, overall and according to peri-implantitis status and univariate models for moderate-severe peri-implantitis (mixed criterion).*

| **Variable** | **Overall**  **N = 511** | **Peri-implantitis status** | | **OR** | **95% CI** | P value |
| --- | --- | --- | --- | --- | --- | --- |
|  |  | **No peri-implantitis (N = 334)** | **Peri-implantitis (N = 177)** |  |  |  |
| Jaw, N (%)  Maxilla  Mandible | 302 (59.1)  209 (40.9) | 207 (62.0)  127 (38.0) | 95 (53.7)  82 (46.3) | *Ref*  1.70 | 1.04-2.79 | 0.034 |
| Position, N (%)  Anterior (canine-canine)  Posterior | 115 (22.5)  396 (77.5) | 79 (23.6)  255 (76.4) | 36 (20.3)  141 (79.7) | *Ref*  1.54 | 0.85-2.78 | 0.155 |
| Reason for tooth loss, N (%)  Caries  Periodontitis  Trauma, fracture  Agenesia | 284 (55.6)  162 (31.7)  50 (9.8)  15 (2.9) | 180 (53.9)  107 (32.0)  37 (11.1)  10 (3.0) | 104 (58.8)  55 (31.1)  13 (7.3)  5 (2.8) | *Ref*  0.91  0.82  1.32 | 0.44-1.88  0.29-2.36  0.26-6.74 | 0.796  0.717  0.738 |
| Implant Brand, N (%)  Nobel Biocare  Biomet 3i  Others | 467 (91.4)  33 (6.5)  11 (2.1) | 305 (91.3)  20 (6.0)  9 (2.7) | 162 (91.5)  13 (7.3)  2 (1.2) | *Ref*  1.25  0.46 | 0.44-3.57  0.07-2.94 | 0.676  0.411 |
| Type of prosthetic restoration, N (%)  Single crown  Bridge  Full-arch fixed restoration  Overdenture | 215 (42.1)  225 (44.0)  64 (12.5)  7 (1.4) | 141 (42.2)  150 (44.9)  38 (11.4)  5 (1.5) | 74 (41.8)  75 (42.4)  26 (14.7)  2 (1.1) | *Ref*  0.92  1.23  0.65 | 0.50-1.67  0.48-3.19  0.06-6.72 | 0.775  0.666  0.717 |
| Prosthesis retention, N (%)  Cemented  Screw-retained | 201 (39.3)  310 (60.7) | 121 (36.2)  213 (63.8) | 80 (45.2)  97 (54.8) | *Ref*  0.54 | 0.29-0.99 | 0.049 |
| Implant-abutment connection, N (%)  External  Internal  Conical | 314 (61.4)  149 (29.2)  48 (9.4) | 201 (60.2)  104 (31.1)  29 (8.7) | 113 (63.8)  45 (25.4)  19 (10.7) | *Ref*  0.99  1.42 | 0.55-1.81  0.87-2.52 | 0.989  0.095 |
| Platform Switching, N (%)  No  Yes | 444 (86.9)  67 (13.1) | 290 (86.8)  44 (13.2) | 154 (87.0)  23 (13.0) | *Ref*  1.29 | 0.62-2.72 | 0.498 |
| Buccal soft tissue phenotype, N (%)  Thin  Thick | 75 (14.7)  436 (85.3) | 49 (14.7)  285 (85.3) | 26 (14.7)  151 (85.3) | *Ref*  0.92 | 0.45-1.88 | 0.819 |
| Keratinized tissue height (buccal)  0 mm  0-2 mm  >2 mm | 64 (12.5)  194 (38.0)  253 (49.5) | 28 (8.4)  129 (38.6)  177 (53.0) | 36 (20.3)  65 (36.7)  76 (42.9) | *Ref*  0.48  0.39 | 0.22-1.03  0.18-0.84 | 0.060  0.016 |
| Correct implant placement, N (%) (bucco-lingual)  No  Yes | 459 (89.8)  52 (10.2) | 303 (90.7)  31 (9.3) | 156 (88.1)  21 (11.9) | *Ref*  0.51 | 0.22-1.16 | 0.109 |
| Correct implant placement, N (%) (mesio-distal)  No  Yes | 154 (30.1)  357 (69.9) | 95 (28.4)  239 (71.6) | 59 (33.3)  118 (66.7) | *Ref*  0.40 | 0.28-0.57 | <0.001 |
| Accessibility to oral hygiene, N (%)  Proper  Limited | 436 (85.3)  75 (14.7) | 280 (83.8)  54 (16.2) | 156 (88.1)  21 (11.9) | *Ref*  1.36 | 0.63-2.95 | 0.007 |
| Plaque, N (%)  0 sites  1-5 sites  6 sites | 36 (7.0)  307 (60.1)  168 (32.9) | 26 (7.8)  218 (65.3)  90 (27.0) | 10 (5.6)  89 (50.3)  78 (44.1) | *Ref*  0.90  1.94 | 0.34-2.42  0.67-5.58 | 0.836  0.218 |
| modified Bleeding Index, N (%)  No/spot bleeding  Linear bleeding  Profuse bleeding | 58 (11.3)  123 (24.1)  330 (64.6) | 47 (14.1)  88 (26.3)  199 (59.6) | 11 (6.2)  35 (19.8)  131 (74.0) | *Ref*  2.69  3.48 | 0.91-7.94  1.26-9.66 | 0.072  0.017 |
| Previous treatment at implant level, N (%)  No treatment  OHI  Non-surgical therapy  Surgical therapy  Soft tissue augmentation | 426 (83.4)  62 (12.1)  4 (0.8)  11 (2.1)  8 (1.6) | 268 (80.2)  51 (15.3)  3 (0.9)  6 (1.8)  6 (1.8) | 159 (89.3)  11 (6.2)  1 (0.6)  5 (2.8)  2 (1.1) | *Ref*  0.32  0.52  1.10  0.40 | 0.11-0.96  0.02-12.8  0.20-5.94  0.05-3.28 | 0.043  0.689  0.914  0.390 |
| Prosthesis gap, N (%)  Yes  No | 110 (21.5)  401 (78.5) | 65 (19.5)  269 (80.5) | 45 (25.4)  132 (74.6) | *Ref*  0.46 | 0.82-2.62 | 0.200 |
| Emergence angle, N (%)  ≤ 30°  > 30° | 345 (67.5)  166 (32.5) | 225 (67.4)  109 (32.6) | 120 (67.8)  57 (32.2) | *Ref*  0.95 | 0.57-1.59 | 0.851 |
| Cantilever, N (%)  Yes  No | 19 (3.7)  492 (96.3) | 6 (1.8)  328 (98.2) | 13 (7.3)  164 (92.7) | *Ref*  5.27 | 1.48-18.8 | 0.010 |
| Implant survival (years), N (%)  ≥ 10 years  < 10 years | 345 (67.5)  166 (32.5) | 204 (61.1)  130 (38.9) | 141 (79.7)  36 (20.3) | *Ref*  2.79 | 1.47-5.31 | 0.002 |

*Abbreviation: N; number; SD, standard deviation; OHI, oral hygiene instruction*
